# Developing an Individual Glucose Prediction Model Using Recurrent Neural Network

**DOI:** 10.3390/s20226460

**Published:** 2020-11-12

**Authors:** Dae-Yeon Kim, Dong-Sik Choi, Jaeyun Kim, Sung Wan Chun, Hyo-Wook Gil, Nam-Jun Cho, Ah Reum Kang, Jiyoung Woo

**Affiliations:** 1 Department of Internal Medicine, Soonchunhyang University Cheonan Hospital, Cheonan 31151, Korea; c99851@schmc.ac.kr (D.-Y.K.); waan@schmc.ac.kr (S.W.C.); hwgil@schmc.ac.kr (H.-W.G.); chonj@schmc.ac.kr (N.-J.C.); 2 Department of Medical Science, Soonchunhyang University, Asan 31538, Korea; milkymy@sch.ac.kr; 3 Department of Big Data Engineering, Soonchunhyang University, Asan 31538, Korea; kimym38@sch.ac.kr; 4 SCH Convergence Science Institute, Soonchunhyang University, Asan 31538, Korea

**Keywords:** continuous glucose monitoring, diabetic inpatient, glucose prediction model, deep learning

## Abstract

In this study, we propose a personalized glucose prediction model using deep learning for hospitalized patients who experience Type-2 diabetes. We aim for our model to assist the medical personnel who check the blood glucose and control the amount of insulin doses. Herein, we employed a deep learning algorithm, especially a recurrent neural network (RNN), that consists of a sequence processing layer and a classification layer for the glucose prediction. We tested a simple RNN, gated recurrent unit (GRU), and long-short term memory (LSTM) and varied the architectures to determine the one with the best performance. For that, we collected data for a week using a continuous glucose monitoring device. Type-2 inpatients are usually experiencing bad health conditions and have a high variability of glucose level. However, there are few studies on the Type-2 glucose prediction model while many studies performed on Type-1 glucose prediction. This work has a contribution in that the proposed model exhibits a comparative performance to previous works on Type-1 patients. For 20 in-hospital patients, we achieved an average root mean squared error (RMSE) of 21.5 and an Mean absolute percentage error (MAPE) of 11.1%. The GRU with a single RNN layer and two dense layers was found to be sufficient to predict the glucose level. Moreover, to build a personalized model, at most, 50% of data are required for training.

## 1. Introduction

Diabetes is a major risk factor for patients suffering from various diseases including cardiovascular disease [1]. The socioeconomic losses resulting from acute and chronic complications and the burden of medical expenses for individual patients are enormous [2,3]. It is also widely known that diabetic patients experience longer hospitalization periods and have higher mortality rates from other diseases than general patients without diabetes [4,5]. Therefore, it is critical to thoroughly monitor, predict, and regulate blood glucose in diabetic patients.

Most previous studies on the prediction of blood glucose were conducted on outpatients, and most of these patients were those with Type-1 diabetes [6,7,8,9,10,11]. As inpatients are hospitalized owing to various diseases, have different personal characteristics, and show dynamic changes during hospitalization, the fluctuation of blood glucose in these inpatients is more severe than in outpatients, and it is, therefore, difficult to predict blood glucose. Inpatients have higher risk of hyperglycemia and hypoglycemia for reasons such as instability of vital signs due to various diseases, stress, and inflammation, immune responses, and drugs administered for treatment [12,13].

The role of medical professionals, especially doctors, is the key to controlling blood glucose in hospitalized patients. On the basis of the blood glucose value measured in the patient’s ward, doctors empirically predict changes in blood glucose levels and adjust drugs including insulin. However, this system is very inefficient, particularly because the number of medical professionals who specialize in diabetes is small compared to the number of diabetic patients. This issue needs to be resolved because excessive labor of medical personnel is demanded in such cases.

Recently, automated systems using artificial intelligence (AI) were investigated in various engineering fields, and the results have been used as a basis for real-life considerations. Medical specialists have attempted to combine AI with medicine in line with this trend of the times. However, the amount of information generated by the human body is close to infinity, and signals from various organs are complexly affected. In the field of AI, the analysis of limited information can lead to an incorrect result; subsequently, the application of an incorrect result is applied to real patients, it can lead to fatal results. Therefore, research involving the application of AI in the medical field is lagging behind that in other fields. However, the utilization of AI is a trend that is difficult to resist, and it is likely to be used in the medical field while reducing errors. We, therefore, believed that the prediction of blood glucose was appropriate as a starting point for the study because such an avenue is less likely to cause immediate harm to patients. Our work has the following motivations. The research uses continuous glucose monitoring for Type-2 patients who make up a major portion in diabetics. Continuous glucose monitoring (CGM) has been performed for Type-1 patients who are usually children and need parents’ care. Parents who have a role to monitor their children’s glucose level often use a CGM device. Thus, previous works for glucose prediction were focused on Type-1 diabetes. Type-2 patients, who are usually adults, are somewhat able to control their glucose level and are reluctant to use CGM devices which are expensive. However, when the patients are in the hospital, their glucose level should be monitored because of its high variability. The prediction model will eventually help to automatically control the amount of insulin according to hypoglycemia, hyperglycemia, and nocturnal glucose. With these motivations, we collected blood glucose data from hospitalized patients with Type-2 diabetes via a CGM system. We then developed a personalized prediction model that applies a recurrent neural network (RNN) to the blood glucose data collected through the CGM. For this, we examined various types of models with different model architectures and hyperparameters, designed data frames to be fitted to the prediction model, and performed experiments. Our work has a contribution in three folds. First, we performed the continuous glucose monitoring for Type-2 inpatients. Second, we found the best architecture for the RNN-based model to capture the patterns from the time-series of the continuous glucose level. Third, our RNN-based model showed comparative results with outpatients with Type-1.

## 2. Related Work

Several studies on blood glucose prediction have been conducted. Type-2 diabetes accounts for 90% of all diabetes, but there are few related studies. It is difficult to predict blood glucose in the case of hospitalized patients with Type-2 diabetes since blood glucose fluctuations are high. There are a few studies on outbreak prediction in hospitalized patients with Type-2 diabetes. We investigated studies using AI on the glucose level prediction using CGM data in patients with Type-1 diabetes and on the outbreak prediction for Type-2 diabetes.

Most of the blood glucose prediction studies used CGM data and diet information of Type-1 diabetes patients [6,7,8,9,10,11,14,15,16,17,18,19,20,21,22,23,24]. Pérez-Gandía et al. [6] predicted blood glucose after 15, 30, and 45 min after a given time using an artificial neural network (ANN) model and used the CGM data from 15 Type-1 diabetic patients. The root mean square error (RMSE) was used to evaluate the accuracy of the model in a glucose prediction study, and a continuous glucose error grid analysis (CG-EMA) and mean absolute difference percentage (MAD) analysis were also conducted. In this study, RMSE values of 10, 18, and 27 mg/dL were found corresponding to 15, 30, and 45 min, respectively.

Pappada et al. [7] collected and analyzed data on 27 Type-1 diabetic patients using the CGM data. They used a feedforward neural network (FNN) model to predict blood glucose levels 75 min after a given time. The FNN model yielded an RMSE of 43.9 mg/dL and MAD of 22.1. A majority of the predicted values (92.3%) were contained within zones A (62.3%) and B (30.0%) of the CG-EMA and were therefore considered clinically acceptable.

Zarkogianni et al. [8] evaluated four glucose prediction models for patients with Type-1 diabetes via the CGM data. The four models were based on an FNN, a self-organizing map (SOM), a neuro-fuzzy network with wavelets as activation functions (WFNN), and a linear regression model (LRM). Each model predicted blood glucose 30, 60, and 120 min after a given time, and the predictive performance was compared. The model using SOM showed better results than using the FNN, WFNN, and LRM. The RMSE values for 30, 60, and 120 min corresponded to 12.29, 21.06, and 33.68 mg/dL. In addition, according to the CG-EMA, the model using SOM showed relatively high accuracy for not only euglycemia but also hypoglycemia and hyperglycemia.

Mhaskar et al. [9] analyzed clinical data from 25 Type-1 diabetic patients using the CGM data and predicted blood glucose 30 min after a given time using deep neural networks (DNNs), especially deep convolutional neural networks (DCNNs). While most studies dealt with one patient at a time, these scholars considered the data of only a certain percentage of patients in the dataset as the training data and tested the remainder of the patients. Based on the CG-EGA, for 50% of the data used for training, the percentage of accurate prediction and predictions with benign consequences was 96.43% in the hypoglycemic range, 97.96% in the euglycemic range, and 85.29% in the hyperglycemic range.

Sun et al. [10] trained and tested on 26 datasets from 20 Type-1 diabetic patients using long-short term memory (LSTM) and bi-directional LSTM (Bi-LSTM)-based deep neural network. RMSE values for 15, 30, 45, 60 min were 11.633, 21.747, 30.215, 36.918 mg/dL.

Li et al. [11] analyzed the data from 10 patients with Type-1 diabetes and proposed a deep learning algorithm using a multi-layer convolutional recurrent neural network (CRNN) model. RMSE values for 30 and 60 min were 21.07 and 33.27 mg/dL.

Sparacino et al. [14] predicted the hypoglycemic threshold 20–25 min ahead using a first-order autoregressive (AR) model. They collected glucose for 48 h using a CGM device that was monitored every 3 min on 28 Type-1 diabetes volunteers. The RMSE values are 18.78 and 34.64 mg/dL for 30 and 45 min. They showed that even using this simple method, glucose can be predicted in advance.

Mougiakakou et al. [15] developed models for simulating glucose–insulin metabolism. They used CGM data, insulin, and food intake from four children with Type-1 diabetes as the input to the models. The FNN, RNN/Free-Run (FR), and RNN/Teacher-Forcing (TF) models were compared. RNN/FR ignores glucose measurements available during training. RNN/TF replaces the actual output during training with the corresponding available glucose measurement. They have shown that models using RNN trained with the Real-Time Recurrent Learning (RTRL) algorithm can more accurately simulate metabolism in children with Type-1 diabetes.

Turksoy et al. [16] used linear ARMAX models composed of autoregressive (AR), moving average (MA), and external inputs (X). They proposed the hypoglycemia alarm system for preventing hypoglycemia before it happens with glucose concentration, insulin, and physical activity information. CGM data from 14 Type-1 diabetic young adults were used for the prediction and alarm algorithm. In the real-time case, the optimal performance was the RMSE value of 11.7 mg/dL for 30 min.

Zecchin et al. [17] proposed a prediction algorithm combined with an NN model with a first-order polynomial extrapolation algorithm for short-time glucose prediction using CGM data and carbohydrate intake. They monitored 15 Type-1 diabetic patients for 7 days using a CGM system that returns glucose values every minute. They showed that using carbohydrate intake information improves the accuracy of short-term prediction of glucose concentration.

Robertson et al. [18] performed blood glucose level (BGL) prediction using an Elman RNN model with the AIDA freeware diabetes simulator. CGM data, meal intake, and insulin injections were used as inputs to the model. The most accurate predictions in their study were in the nocturnal period of the 24-h day cycle. The results were an RMSE value of 0.15 ± 0.04 SD mmol/L for short-term (15, 30, 45, and 60 min) and an RMSE value of 0.14 ± 0.16 SD mmol/L for long-term predictions (8 and 10 h).

Georga et al. [19] proposed the application of random forests (RF) and RReliefF feature evaluation algorithms on Type-1 diabetes data. RReliefF is a feature ranking algorithm for regression problems. They evaluated several features, CGM data, food intake, plasma insulin concentration, energy expenditure, and time of the day, extracted from medical and lifestyle self-monitoring data. They showed that the information on physical activities is able to improve performance.

Jaouher et al. [20] proposed an ANN-based method for predicting blood glucose levels in Type-1 diabetes using CGM data as inputs. They investigated real 13 Type-1 diabetic patients to validate their ANN model. The RMSE values were 6.43 mg/dL, 7.45 mg/dL, 8.13 mg/dL, and 9.03 mg/dL for 15, 30, 45, and 60 min. They suggested that using only CGM data as inputs and limiting human intervention for improving the quality of life of Type-1 diabetic patients.

Martinsson et al. [21] proposed an RNN model to predict blood glucose levels. CGM data from 6 Type-1 diabetic patients (OhioT1DM [22]) were used for training and evaluation. OhioT1DM dataset consists of blood glucose level values for two men and four women. The RMSE values were 18.867 mg/dL and 31.403 mg/dL for 30 and 60 min. They pointed out that larger data sets and standards are needed.

Aliberti et al. [23] developed a patient-specialized prediction model based on LSTM. They used OhioT1DM dataset for training and validation. Among the 6 patients, the RMSE values of the patient with the best predicted result were 11.55 mg/dL, 19.86 mg/dL, 25 mg/dL, and 30.95 mg/dL for 30, 45, 60, and 90 min. The worst results were 11.52 mg/dL, 19.58 mg/dL, 27.67 mg/dL, and 43.99 mg/dL for 30, 45, 60, and 90 min.

Carrillo-Moreno et al. [24] proposed an LSTM model using historical glucose levels, insulin units, and carbohydrate intake data. They used CGM data from 3 Type-1 diabetic patients and created 12 models with various combinations of patient-specific, prediction horizon (PH), LSTM layers, and the number of neurons for performance comparison. They found that the predictor with a PH of 30 min is the best performance.

As far as we know, no study on blood glucose prediction has been conducted in patients with Type-2 diabetes. The current state is that the prediction model for Type-2 diabetes outbreak has been proposed [25,26]. Wu et al. [25] proposed a new model using Weka open source machine learning software to predict Type-2 diabetes mellitus. Personal health data and medical examination results from 768 patients were used to train and test whether patients had diabetes. The number of times pregnant, plasma glucose concentration at 2 h in an oral glucose tolerance test, diastolic blood pressure, triceps skin fold thickness, 2-h serum insulin, body mass index, diabetes pedigree function, age, class variable were used as features. Diabetes prediction accuracy is 96%.

Kazerouni et al. [26] proposed Type-2 diabetes mellitus prediction using data mining algorithms based on the long-noncoding RNAs’ (lncRNA) expression. They used data from 100 Type-2 diabetic patients and 100 healthy individuals. As features, 6 lncRNA expressions, gender, age, weight, height, BMI and fetal bovine serum (FBS) were used. They applied four classification models of K-nearest neighbor (KNN), support vector machine (SVM), logistic regression, and ANN and compared their diagnostic performance. Logistic regression and SVM showed 95% of mean area under the receiver operating characteristic (ROC) curve. These studies are in patients with Type-2 diabetes, but differs from our study, which predicts blood glucose with CGM data.

Previous works developed from statistical models, AR species to deep learning, RNN. However, they limit the experiment to Type-1 diabetics, outpatients, and the limited number of patients and the short period. In this work, we set the experiment venue to an in-hospital patient with Type-2 diabetes.

## 3. Materials and Methods

### 3.1. Subjects and Database

The evaluation of the glucose prediction model was performed using data collected via the Dexcom G5(R) Mobile CGM (Dexcom, Inc., San Diego, CA, USA) from 20 Type-2 diabetic patients. The Dexcom G5 is composed of a sensor, transmitter, and mobile app on a smart device and allows patients to view real-time continuous sensor glucose readings on their own every 5 min for up to 7 days (288 samples per day and 2016 samples per week). The patients considered had been admitted to Soonchunhyang University Cheonan Hospital between July 2019 and March 2020. All the patients met the following inclusion criteria: they had to be ≥20 and <70 years of age, and they had to be inpatients suffering from diabetes, inclusive of intensive care unit (ICU) patients. The patients were made to wear the Dexcom G5 for at least 3–7 days during hospitalization.

### 3.2. Research Framework

**Figure 1:**
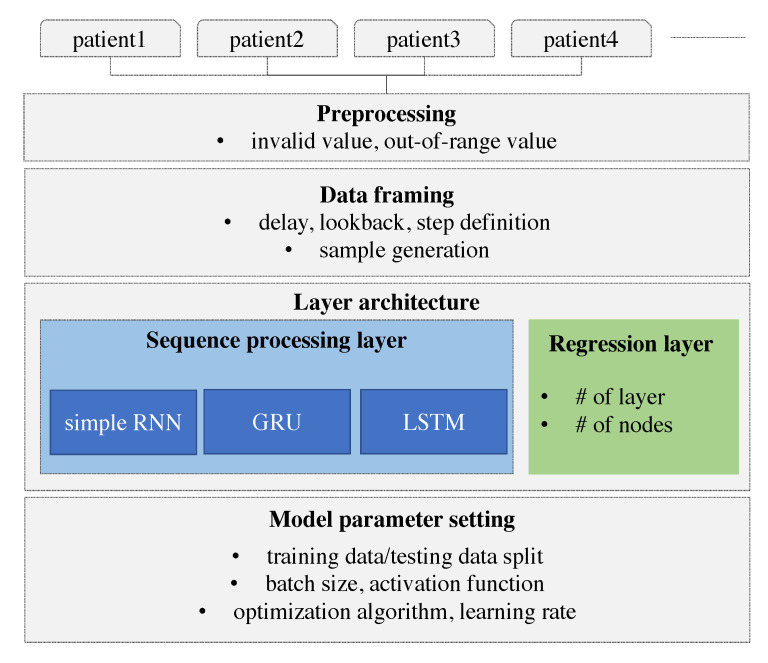
Research framework.

Herein, we develop a personalized model using deep learning. The model is designed to learn and derive patterns from the individual patients’ time-series and predict the future value based on the personalized model. The model is built using an RNN and the results are compared with those of different types of RNN. We adopt especially RNN that can consider the sequence of data. The time-series of individual glucose data is collected and the individual glucose data are preprocessed. As a remote device may lose the Bluetooth signal, missing values can be resulted in. These missing values and out-of-range values are also removed. The device does not record the value over 400 or below 60. We consider three different types of RNNs that differ on the basis of how the sequence is processed. These RNN models are specified with the number of layers and the number of nodes in each layer. The hyper-parameters of the RNN model such as optimization algorithm, activation function, batch size, and the learning rate are explored to derive the best model. To prepare the data to be fitted to the model, the data framing process is performed specifying the lookback, delay, and the length of the target value. A sample is generated with the length of the lookback and the target value is set with the delay from the last value of the input sample. More specifically, the lookback indicates how many observations in previous time steps will go back to form an input sample and the delay indicates how many time steps in the future the target have to be. Our research framework is demonstrated in Figure 1.

### 3.3. Prediction Algorithms

Recurrent neural networks (RNNs) have recently shown promising results in many machine learning tasks for time-series data. Especially when input and/or output are of variable length, RNN is easy to be applied [27].

The RNN processes sequential glucose values in an iterative manner and maintains its state by means of transforming previous sequences. This state remembers the manner in which the previous values affect the prediction system.

**Figure 2:**
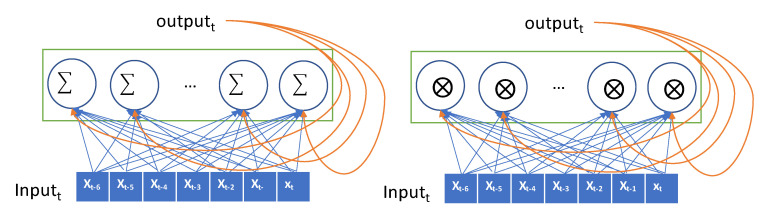
Conceptual description of a recurrent neural network (RNN) (Simple RNN on the left and gated recurrent unit (GRU) on the right).

The input is encoded as a 2D tensor of size (timesteps, input_features) and the current input is iteratively fed to the RNN over the time steps. The current output is remembered as a state and fed to the RNN combined with the subsequent input. In Figure 2, the recurrent layer takes the input and generates the output by combining the input, and it then hands the current output to the next RNN node as a state. In our data, the lookback period is set to 7, the step size is 1, and the delay is 6. The seven nodes in the RNN layer process the input data sequentially, exploit the temporal order of the input, and generate a single target value that is the glucose value, after 30 min. The values in the sequence that are as long as the lookback are transferred into the RNN node and transformed as an output. This output is forwarded to the next RNN node as a state. Thus, the effect of the first value in the sequence can be diminishing as the input value is processed in the sequence. The values at the end of the sequence have a greater impact on the output than the values at the start of the sequence. This causes that the error in the output have a high impact in updating the weights in recent inputs, but the output error has little impact in updating the weights in the former input. This problem is called the vanishing gradient problem. GRU and LSTM models were proposed herein to overcome this vanishing gradient problem. The GRU has a gate layer that can forget the current input value when it is needed in the RNN layer [28]. When the current input does not help to predict the output, the RNN layer only retains the previous state. This retains previous inputs and reduces the diminishing gradient problem. While a simple RNN model adds up the current input and previous hidden state, GRU takes xor operation with the gate that decides whether to choose the current input or the hidden state as shown in Figure 2.

**Figure 3:**
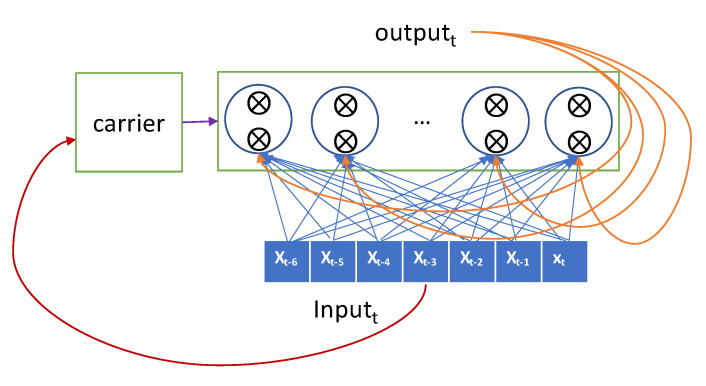
Conceptual description of long-short term memory (LSTM).

The more sophisticated one, the LSTM model, has a carrier that carries the previous input over the lookback-length time steps [29]. This carrier retains the previous input seen so far as original forms and hands them over to an RNN node when required. The current input is forwarded selectively to the current RNN node to generate the output, and it is also forwarded selectively to the hidden state that will be connected to the input in the next iteration. The gate decides whether it passes the input or not. The LSTM advances the GRU by adopting two gates and a carrier as shown in Figure 3.

The LSTM unit is known to work well on sequence-based tasks with long-term dependencies. GRU is newly introduced and generates a comparative performance to LSTM. According to Chung’s work [27], GRU showed the superior performance over LSTM. LSTM and GRU can bypass units and thus remember for longer sequences, but this remembering is computationally expensive. Like the LSTM unit, the GRU has gating units that modulate the flow of information inside the unit; however, it does not have a separate memory cell unlike the LSTM unit. Thus, GRU is more computationally effective than LSTM.

### 3.4. Data Framing

**Figure 4:**
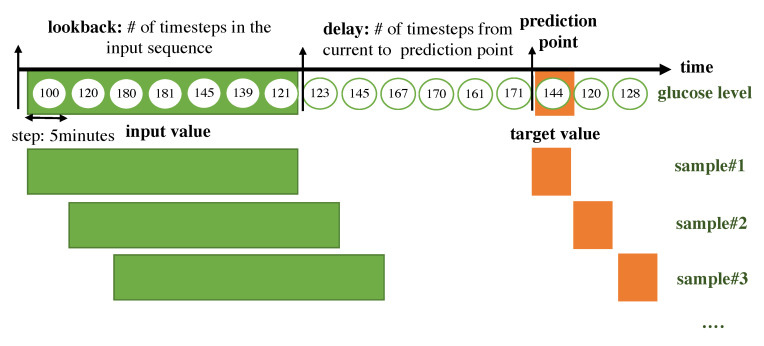
Overall description of how the data are framed to be fitted to the RNN model.

The RNN for a time-series takes its input as a 2D form. The time steps are chucked according to the lookback period. We split the input data as long as the lookback period, which indicates how many previous values should be seen together in the RNN module and we consider the set of timesteps within the lookback as a sample. Every sample is generated with a sliding window with a step size of 1. The target value that the algorithm should predict is set with a delay, which represents the number of timesteps from the current to prediction points. A sample is composed of a set of previous values within the lookback period and a target value. An overall description of how the data are framed to be fitted to the RNN model is shown in Figure 4.

1. lookback: Observations will go back and be seen.
2. sampling rate: Observations will be sampled at two data points per hour.
3. delay: A target will be predicted after a delay in the future.
4. windowing step: A sample is generated at every time step.

**Figure 5:**
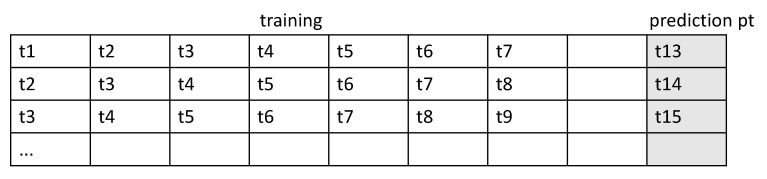
Form of the input sample.

The form of the input sample is shown in Figure 5.

### 3.5. Model Setup

After the sample generation, we design various scenarios to achieve the best model for the glucose prediction. We vary the sample split ratio and various model architectures with different hyper-parameters and with/without the event input. We first compare different types of RNNs as mentioned in the subsection of Prediction Algorithms. Second, we test the model architectures by increasing the capacity of the network and we change the number of stacks. In the two stack levels of the RNN, the RNN layer at the upper level takes input corresponding to the state of the RNN layer at the lower level. In this case, the RNN layer at the lower level returns the sequence of the state instead of generating an output at the end of the time step within a lookback. A bi-directional RNN is also explored. While the RNN processes the time steps of input sequences in time order, the bi-directional RNN takes inputs in time order and also takes input in reverse time order. Revision completely changes the representations of input sequences; this may improve the performance in certain cases, particularly in the language domain.

Third, we check for the optimal size of the training samples. Among the entire samples, the ratio of training and validation is set. The more the training samples are used, the greater the algorithm improvement; however, for practical deployment of an individual prediction model, the algorithm should be built with a minimal number of training samples. We vary the lengths of the training samples and conduct tests to determine how many samples are required to build the model.

## 4. Data Statistics

The demographics of the patients enrolled in our experiment are shown in Table 1. The construction of the database was approved by SoonChunHyang University Hospital Cheonan Institutional Review Board (SCHCA IRB Protocol Number: SCHCA 2019-11-048). The glucose levels were distributed as indicated in Table 2. The highest value was 400, the lowest value was 60, and the average level was 193.1 for all patients.

## 5. Results

First, we compared the RNN variants with the baseline architecture of an RNN layer and two fully connected layers. The RNN adds memory ability to input sequences, and the fully connected layer combines the RNN nodes all together and generates a target value.

The batch size refers to the number of training samples in one forward pass of an RNN before a weight update. The epochs refer to the total number of forward pass iterations. Typically, more epochs improve the model performance unless overfitting occurs, at which time the validation accuracy/loss will not improve. The batch size was set to 20 in our experiment, and rmsprop was adopted as an optimization algorithm.

The baseline models with different types of RNN layers were tested when 70% of the samples were used for training and 30% of samples are used for testing.

### 5.1. Algorithm Comparison

We tested three different types of RNN algorithms, a simple RNN that takes an input sequentially and iteratively updates an output, a GRU that decides to take a current input for generating output, and an LSTM that embeds a carrier that delivers the former value to the future. In our case, the GRU outperformed the other algorithms, as shown in Table 3. This result implies that integrating to a single value would be better rather than remembering the initial values.

### 5.2. Model Architecture Comparison

For the GRU, we tuned the model architecture by adding the activation functions in the RNN layer. This however lowered the performance. For the architecture without the activation function, the bi-directional layer and two layers were tested. The best-performing model was found to have an architecture wherein one layer was without an activation function. We tested a tanh activation function in the recurrent layer and a ReLU function in the dense layer. We also tested as to whether stacking a recurrent layer increased the representational power of the network. The bidirectional recurrent layer reads data in the opposite direction. As the network capacity increases with two layers and the reverse layer, the performance decreases. The results indicate that remembering the reverse sequences is not helpful, and more abstract patterns from sequences are not necessary for glucose prediction; all these results are listed in Table 4.

### 5.3. Training Sample Size Comparison

Next, we varied the ratio of training over the testing data period and the results of which are displayed in Table 5. As less data were provided to the training phase, the accuracy decreased when the training samples constituted less than 50% of the total samples. This implies that the model development should be updated until at most 50% of the total data are provided.

### 5.4. Model Improvement

In this section, we examined the hyper-parameters together with setting GRU and performed the sensitivities analysis. To improve the performance, we conducted tests on shuffling and increasing the batch size. We found that shuffling, whereby input samples are randomly selected instead of being composed within a batch sequentially, was effective in yielding a performance improvement. In addition, a larger batch size improved the performance. The heterogeneous samples rendered the model unstable when they were fed to the model one by one. We were able to improve the performance from 22.26 to 21.46 in terms of the RMSE.

We changed the optimization algorithm from RMSprop to Adamax, which caused the performance decrease. We also changed the number of epochs. The lower epoch and higher epoch generated the lower performance. The various attempts explored for model improvement are shown in Table 6.

## 6. Discussion

**Figure 6:**
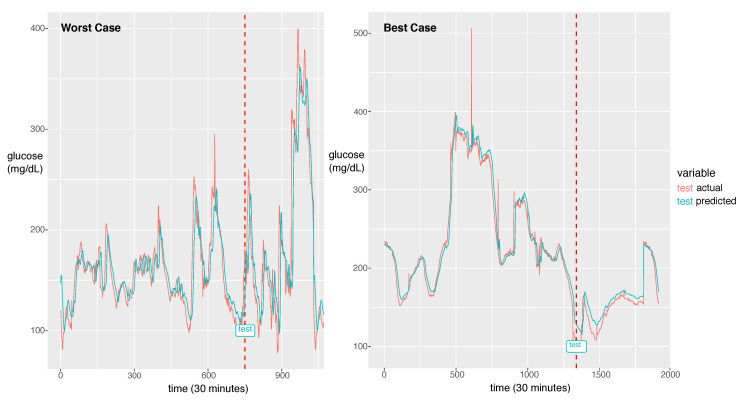
The worst case in the first panel and the best case in the second panel. The dotted line is the start time of testing. The red line is for the reference value and the green line is for the prediction value.

For the best model, the RMSE was 21.46 and the MAPE was calculated as 11.11%. The actual glucose value and the prediction value obtained using the best model for a patient with a 7:3 sampling split are shown in Figure 6.

The model could learn the sharp peak in the training phase and generate a larger error in the sharp peak zone compared to the smooth zone. The glucose prediction is expected to be sensitive to low values (hypoglycemia), but our model is not sensitive to high values (hyperglycemia). Thus, this problem can be overlooked when this algorithm is implemented in practice. As a future work, we will investigate the solution for the large error in hyperglycemia.

In Figure 6, we demonstrate two cases of the best and the worst of the prediction performance. We could notice a big difference between the two. The case that the fluctuation with high frequency exhibits bad performance had an RMSE of 44.15 and the best case of the fluctuation with low frequency exhibits the best performance with an RMSE of 8.35. As a future work, we will update the model to capture the high frequent fluctuation. A probable solution is to train the model using the change rate of glucose as data instead of the glucose value itself. Another difference between the two cases is that the worst case has a much shorter experimental period than the best case. From this point of view, to achieve a good performance, enough training data should be provided. The other solution would be data augmentation to provide enough data for the fluctuation with high frequency.

**Figure 7:**
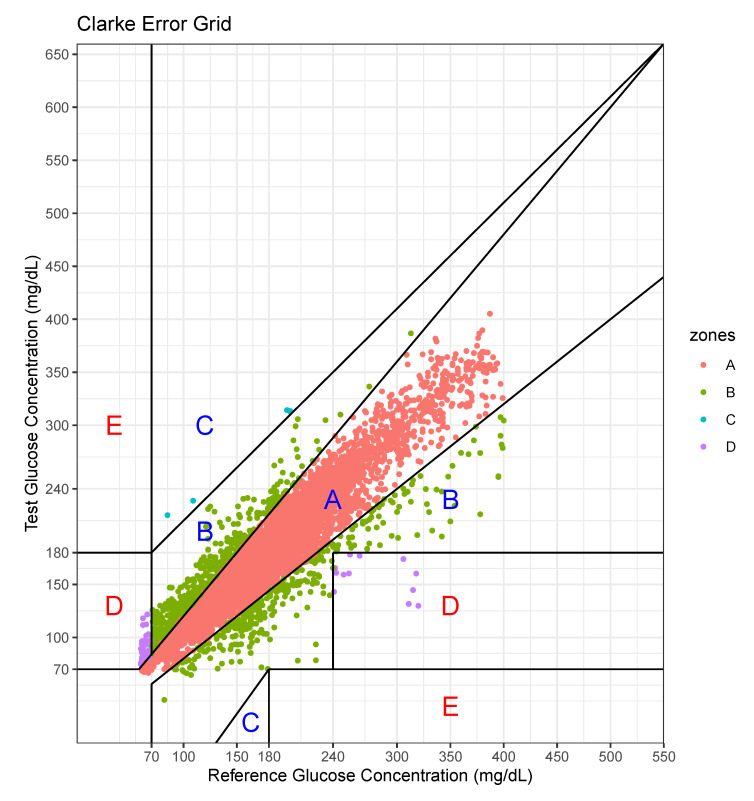
Error analysis with two axes—actual value and predicted value.

We performed an error analysis for the predicted values, as shown in Figure 7. The error analysis follows the Clarke error grid lines according to the criteria [30]. This graph confirms the very good clinical agreement between measured and predicted glucose values. Most of predicted values were located in the A zone. Occasional pairs of points fall in the B zone which is the slight overestimation/underestimation zone. Only a few points fall in the erroneous C and D zones. No point falls in the E zone which is a significantly erroneous zone. The percentage of data points in each zone is shown in Table 7.

For this case, the model tended to overestimate the glucose level, especially at low levels of glucose.

In hospitals, the meals are served regularly and other food is prohibited. However, patients can take meals at different times and every person’s daily caloric intake is individual. Furthermore, the intake of other food is hard to be tracked unless patients voluntarily record their meal intake. Thus, in reality, it is difficult to record the exact event information. The event information including meal and insulin inject are important, but we could not afford to collect these data. However, our model is still working due to the following reasons. We collected the data from a hospital, so the same condition is applied to all patients in our experiments. Even in the absence of the event information, the model still can learn the sequence patterns reflecting event effects from the past history including the results of the event. Second, even if the event information becomes available, the stochastic event cannot be incorporated into the forecasting model because the model is required to predict the stochastic event as well. Only periodically deterministic events can be employed in the model because this information can be recorded in the input prior. Nevertheless, the event information would increase the model performance. As a future work, we will utilize the predefined and circadian event such as the meal intake, medicine intake, shot injection and treatment. In future work, we will further develop the model, incorporating more information regarding aspects such as insulin doses and food intake, which significantly affect the glucose level. Thus far, we have not recorded such information, but we plan to collect these details. Second, we developed an individual model that uses the patient’s own data. The number of patients involved for data collection was limited to 20, so it was difficult to leverage others’ data. However, when a large-scale data set becomes available, all patient glucose data can be used as the input. Then, the input will be in the form of vectors composed of patients’ own glucose data and others’ glucose data. The drawback in the current work, however, is that a significant portion of individual data are required for prediction; however, this can be resolved when the model exploits other patients’ data. Third, we will employ more advanced models such as a hybrid model incorporating a CNN and an RNN, a 1D CNN for a long sequence, and an attention-based model for achieving better performance. Dendritic neuron model considering the nonlinearity of synapses can be a good candidate for improving performance [31,32]. Neural network models including RNN or CNN only consider the linearity of synapses in the model. We will study how to incorporate Dendritic neuron model and RNN to derive the sequence pattern based on the nonlinearity.

## 7. Conclusions

In this study, we proposed a personalized glucose prediction model using deep learning for hospitalized patients suffering from Type-2 diabetes. Currently, medical personnel check blood glucose and control insulin doses, and they mainly rely on their domain knowledge of the glucose levels measured in the ward. To reduce the labor of doctors, who are few in number when compared to diabetic patients, and the doctors’ biases with regard to decision-making, AI can be deployed to assist medical personnel. We employed a deep learning algorithm, specially an RNN consisting of a sequence processing layer and a regression layer for the glucose prediction. As the sequence processing layer, we tested three types of models that differ with regard to how they remember previous sequences as they are or transformed the previous sequences into a single state value. We designed a prediction model that exploits the past 35 min of glucose values and predicts a value after 30 min. We found that the LSTM and GRU could remember all previous values as they outperformed a simple RNN; overall, the GRU exhibited the best performance in terms of the average RMSE and MAPE. We also found that training samples covering 50% of the hospitalization period data are sufficient to achieve good performance. This implies that the model should be updated until half the hospitalized period passes. A contribution of this work is that we developed a prediction model for Type-2 diabetic patients while most previous studies focused only on Type-1 diabetic patients. In addition, we collected continuous glucose data for hospitalized patients while most previous studies used outpatient data. Finally, we determined the best architecture for a personalized prediction model: a simple with one GRU layer for a sequence processing and two-stacked dense layers for regression.

## Figures and Tables

**Table 1:** Demographics of enrolled patients.

Age Group		Gender	
30–39	3	Female	13
40–49	6		
50–59	4	Male	7
60–69	7		

**Table 2:** Distribution of the glucose levels of patients.

Patient ID	Experiment Days	Max	Min	Average
S001	5.9	350	73	186.3
S002	3.6	400	78	167.61
S003	4.4	400	60	200.73
S004	6.7	334	47	155.02
S005	6.9	347	40	164.45
S006	7.4	400	117	229.4
S007	5.8	301	71	153.78
S008	4.5	398	60	146.41
S009	6.3	506	93	217.22
S010	2.6	400	169	278.03
S011	3.6	400	62	224.44
S012	4.9	372	56	196.72
S013	2.8	324	60	193.54
S014	3.9	288	67	154.98
S015	6.2	403	88	212.51
S016	3.7	289	80	172.45
S017	6.8	399	62	204.75
S018	4.3	399	145	248.36
S019	3.1	275	79	131.22
S020	3.9	400	62	215.09
Total	99.4	400	60	193.1

**Table 3:** Algorithm comparison.

	RNN	GRU	LSTM
RMSE 7:3 with batch size 20	23.43873	22.26309	23.08421

**Table 4:** Model architecture comparison.

Models	RMSE
One layer in sequence processing	22.26309
Bi-directional	22.41404
Two layers	22.5565

**Table 5:** Training sample size comparison.

Train: Test	GRU Performance
7:3	22.26309
6:4	21.79546
5:5	22.11716
4:6	22.29638
3:7	22.57999

**Table 6:** Model improvement results.

Hyper-Parameters	Performance
Batch 50	22.37776
Batch 50 + Shuffling	21.46406
Batch 50 + With Shuffle + Adamax optimization	21.47056
Batch 50 + With Shuffle + RMSprop optimization + 10 epoch	22.45610
Batch 50 + With Shuffle + RMSprop optimization + 30 epoch	24.21941

**Table 7:** Percentage of data points in grid zones.

Zone	A	B	C	D	E
Percentage	87.92	11.11	0.06	0.91	0
